# Effects of Extractives on Dimensional Stability, Dynamic Mechanical Properties, Creep, and Stress Relaxation of Rice Straw/High-Density Polyethylene Composites

**DOI:** 10.3390/polym10101176

**Published:** 2018-10-22

**Authors:** Huanbo Wang, Fazhi Lin, Pingping Qiu, Tian Liu

**Affiliations:** Key Laboratory of Bio-Based Material Science and Technology (Ministry of Education), College of Material Science and Engineering, Northeast Forestry University, 26 Hexing Road, Harbin 150040, China; wanghuanbov@outlook.com (H.W.); lfzxsyx@163.com (F.L.); pingpingqiu95@163.com (P.Q.)

**Keywords:** extractives, composites, dimensional stability, dynamic mechanical properties, creep, stress relaxation

## Abstract

The removal of rice straw extractives increases the interphase adhesion between rice straw and the high-density polyethylene (HDPE) matrix, while eradicating the inner defects of rice straw/HDPE composites. This study investigated the effect of rice straw extractives removal on the dimensional stability (water uptake and thermal expansion), dynamic mechanical properties, creep, and stress relaxation of rice straw/HDPE composites. Cold water (CW), hot water (HW), and 1% alkaline solution (AL) extraction methods were utilized to remove rice straw extractives. Extracted and unextracted rice straws were mixed with HDPE, maleated polyethylene (MAPE), and Polyethylene wax to prepare composites via extrusion. Removal of rice straw extractives significantly improved the dimensional stability, dynamic mechanical properties, and creep and stress relaxation of rice straw/HDPE composites, with the exception of the thickness swelling of the AL/HDPE and the thermal expansion of the rice straw/HDPE composites. HW/HDPE exhibited the best comprehensive performance.

## 1. Introduction

Natural fiber reinforced polymer composites (NFPCs) have been widely utilized in construction, automotive, packaging, window and door frames, furniture, railroad sleepers, gardening items, shelves, aerospace, leisure, and sports industries. NFPCs yield economic, environmental, and social benefits [1,2,3]. Natural fibers have a bright prospect as a substitution of artificial, non-renewable, and undegradable fibers in polymer composites. However, the polarity of typical natural fibers lead to a relatively low compatibility with non-polar polymer matrices (e.g., polyethylene) and insufficient wetted by non-polar polymer matrices [4]. The weak interfacial adhesion between polar natural fibers and non-polar ones lead to a reduction of stress transfer efficiency from the matrix to the reinforcing fibers. A further drawback of the NFPCs is the increased water absorption due to the hydrophilic natural fibers. Water absorption changes the volume of the fibers within the NFPCs and induces internal stresses [5]. The swelling and shrinking of the fibers that surround the matrix decreases the adhesion between the fibers and the matrix, resulting in a significant degradation of the initial properties of the NFPCs [5]. Moreover, water absorption and temperature variation are able to change the macro dimensional of the NFPCs and bring some negative effects to the practical application of the NFPCs.

**Figure 1:**
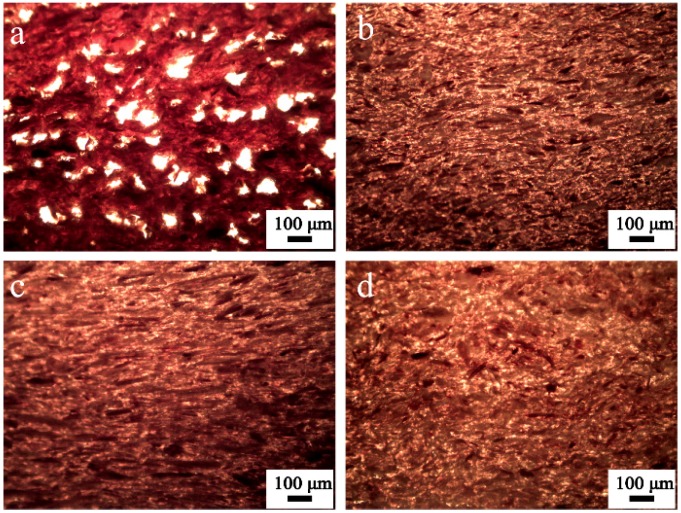
Optical micrographs of the internal state of (**a**) RS/HDPE, (**b**) CW/HDPE, (**c**) HW/HDPE, and (**d**) AL/HDPE.

Natural fibers are mainly composed of cellulose, hemicellulose, and lignin, with a small quantity of extractives, whose content varies depending on their origin. Extractives are non-framework polymers of natural fibers that can be extracted with either neutral organic solvents and/or water [6]. These are complex mixtures of flavonoids, lignans, stilbenes, tannins, inorganic salts, fats, waxes, alkaloids, proteins, simple and complex phenolics, simple sugars, pectins, mucilages, gums, terpenes, starch, glycosides, saponins, and essential oils [2]. At processing temperatures, extractives may tend to concentrate at the surface of natural fibers, thus interfering with the fiber-matrix adhesion of the NFPCs [2,6]. As a residue of rice crops, rice straw (RS) contains a high number of extractives [7,8]. Rice straw has been used as raw material for NFPCs, such as polymer composites [9,10], particleboard [8], and fiberboard [11]. Research regarding the effects of extractives on the rice straw reinforced plastic composites is limited. A large number of voids were found that act as defects in the internal of rice straw/polyethylene (HDPE) composites (Figure 1a) with a high rice straw content (60 wt %), which was in close connection with the die swell. Previous investigations by our group demonstrated that the three methods of extractives removal of rice straw via cold water (CW), hot water (HW), and 1% alkaline (AL) methods change the chemical composition of rice straws and improve the interphase adhesion between the rice straw and the high-density polyethylene (HDPE) matrix. Inner defects (Figure 1b–d) and the die swell phenomenon of RS/HDPE composites are eradicated, and the static mechanical properties of RS/HDPE composites are significantly increased. Extractives removal via CW and HW are simple, low cost, and effective methods compared to the extractives removal via AL. Cellulose and hemicellulose of the natural fibers are the main composition that increase the water absorption of the NFPCs [12,13,14], which can be contributed by their hydroxyl groups [2]. The altered chemical composition of rice straws by extraction might vary the water absorption of the RS/HDPE composites and influence the dimensional stability of the RS/HDPE composites. The altered chemical composition of rice straws, the improved interphase adhesion, and various internal state of the final composites could also show different performances in the practical application of the composites.

To clarify, the dimensional stability (water uptake and thermal expansion), dynamic mechanical properties and creep and stress relaxation behavior of both extracted and unextracted rice straw/HDPE composites were systematically investigated in this study.

## 2. Materials and Methods

### 2.1. Materials

HDPE (5000 s) was obtained from Daqing Petrochemical Co., Daqing, China. Its density was 0.95 g/cm^3^ with a melt flow rate of 0.70 g/10 min. Rice straw was purchased from a local farm in Harbin, China. Rice straw flour that could pass through a 40-mesh was fabricated with a mill in the laboratory. Maleated polyethylene (MAPE) served as a compatibilizer and was supplied by Shanghai Sunny New Technology Development Co., Shanghai, China. MAPE had a melt flow rate of 2 g/10 min and graft ratio of 0.9 wt %. Polyethylene wax was used as lubricant and was purchased from Nanjing Adisi Import & Export Co., Ltd., Nanjing, China. Sodium hydroxide (96% purity) was obtained from the Tianjin DaLu Chemical Reagent Factory, Tianjin, China.

### 2.2. Removal of Rice Straw Extractives

Extractives of rice straw were removed via hot 1% AL solution (T 212 om-02 standard), CW, and HW (T 207 cm-08 standard). The extracted rice straws were named as AL-RS, CW-RS, and HW-RS, respectively. The content of Al, CW, and HW extractives of RS is shown in Table 1.

### 2.3. Preparation of Rice Straw/HDPE Composites

HDPE, MAPE, lubricant, and four dry rice straws (RS, AL-RS, CW-RS, and HW-RS, moisture content <3%) were compounded at specific ratios of 34:4:2:60 wt % in a high-speed mixer (SHR-10A, Zhangjiagang Tongsha Plastic Machinery Company, Zhangjiagang, China) for 10 min at room temperature. A parallel-rotating twin-screw extruder (diameter 518 mm and L/D40, SH30, Nanjing Rubber Machinery Corp., Nanjing, China) was used to press the compounds into pellets at temperatures ranging from 145 to 165 °C. The pellets were then extruded through a single-screw extruder (SJ-45, Nanjing Rubber Machinery Factory, Nanjing, China) into profiles. Both extracted and unextracted rice straw/HDPE composites were individually coded as AL/HDPE, CW/HDPE, HW/HDPE, and RS/HDPE according to extraction method.

### 2.4. Analysis

#### 2.4.1. Compositional Analysis of Rice Straw Flour

The lignin, alpha-cellulose, and ash contents of both extracted and unextracted rice straws were determined in accordance with the Technical Association of Pulp and Paper Industry (TAPPI) methods (APPI T222 om-11, TAPPI T203 cm-99, and TAPPI T211 om-02). A NaClO_2_ treatment [15] was performed to measure the hemicellulose content, which was determined as the difference in value between holocellulose and alpha-cellulose contents. The main chemical composition of the unextracted and extracted rice straw are shown in Table 1.

#### 2.4.2. Fourier Transform Infrared (FTIR) Analysis

A Fourier transform infrared spectrometer equipped with an attenuated total reflectance (ATR) accessory (Nicolet 6700, Thermo Fisher Scientific, Agawam, MA, USA) was used to analysis the hydroxyl groups of both extracted and unextracted rice straw at a resolution of 4 cm^−1^ with 32 scans.

#### 2.4.3. Bulk Density of both Extracted and Unextracted Rice Straw Flour

The method of Pak Sui Lam and Shahab Sokhansanj [16] was used to determine the bulk density of RS, CW-RS, HW-RS, and AL-RS. A funnel with the opening diameter of 1.5 cm was suspended over a cylindrical container with a height-diameter ratio of 1.40. Rice straw flour flowed freely into the cylindrical container from the funnel at a height of 20 cm. Bulk density of rice straw flour was determined by weight/volume and every sample was repeated five times.

#### 2.4.4. Optical Micrograph of the Internal State of Rice Straw/HDPE Composites

The internal states of rice straw/HDPE composites were observed via polarizing microscope (AMART-POL, Chongqing Optec Instrument Co., Ltd., Chongqing, China). Slices with a thickness of 5 μm were cut from the composites using a microtome for the observation.

#### 2.4.5. Water Absorption Test

Water absorption (WA) and thickness swelling (TS) of the AL/HDPE, CW/HDPE HW/HDPE, and RS/HDPE were tested in accordance with ASTM D570. Specimens measuring 25 mm × 25 mm × 4 mm, were oven dried at 60 °C for 24 h. The weight and dimensions of each specimen were measured prior to the test. The specimens were soaked in water at 25 °C for 65 days. The value of WA and TS were calculated following Equations (1) and (2), respectively:

(1) $$\;WA\;\left(\%\right)=\frac{W_{t}-W_{0}}{W_{0}}\times 100\%,\;$$

(2) $$TS\;\left(\%\right)=\frac{T_{t}-T_{0}}{T_{0}}\times 100\%\;,$$

where *W_t_* and *W*_0_ represent the weight (g) of the specimens at specific and initial time, respectively, and *T_t_* and *T_0_* represent the thickness (mm) of the specimens at specific and initial time, respectively.

#### 2.4.6. Thermomechanical Analysis (TMA)

The thermal expansion of the composites was measured using a thermo mechanical analyzer (Q400; TA Instruments Inc., New Castle, DE, USA). The samples (6 mm × 6 mm × 4 mm) were clamped between the probe and the sample table under a force of 0.05 N. Then, they were heated from 30 to 60 °C, cooled to −30 °C, and finally reheated to 30 °C. The heating and cooling rates remained identical at 3 °C/min.

#### 2.4.7. Creep and Relaxation Analysis

Creep and stress relaxation behaviors of the composites were carried out in a single cantilever mode at 30 and 60 °C, respectively, using a dynamic mechanical analyzer (Q800; TA Instruments Inc., New Castle, DE, USA). The samples of both tests measured 35 mm × 12 mm × 4 mm. A load of 2 MPa was applied to the samples for 30 min followed by the release of the load for 30 min in an isothermal creep test. A constant strain of 0.1% was applied to the samples for 30 min for the stress relaxation test, and the change in load was recorded.

#### 2.4.8. Dynamic Mechanical Analysis (DMA)

The dynamic mechanical properties of the composites were measured using a dynamic mechanical analyzer (Q800; TA Instruments Inc., New Castle, DE, USA). Tests of samples measuring 35 mm × 12 mm × 4 mm were performed in a single-cantilever mode with an amplitude of 50 µm and a frequency of 1 Hz. The temperature was swept from −20 to 120 °C at a heating rate of 3 °C/min.

## 3. Results and Discussion

### 3.1. Dynamic Mechanical Analysis

**Figure 2:**
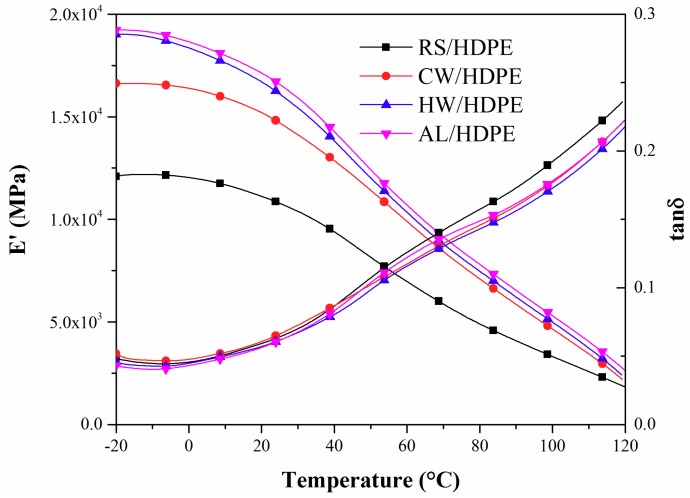
Storage modulus (*E*′) and mechanical damping (tanδ) of RS/HDPE, CW/HDPE, HW/HDPE, and AL/HDPE as a function of temperature.

The storage modulus (*E*′ of both extracted and unextracted rice straw/HDPE composites decreased over the entire measured temperature range. This might be caused by the increased mobility of HDPE chain segments with increasing temperature [14]. AL/HDPE had the highest *E*′, followed by HW/HDPE, CW/HDPE, and RS/HDPE (Figure 2). *E*′ was closely associated with the elastic response of the composites and represented the stiffness of the material. The natural fibers greatly contributed to the NFPCs’ stiffness and its interaction with the matrix also influenced the stiffness of the material [17,18]. Extracted rice straw/HDPE composites had higher *E*′ than unextracted rice straw/HDPE composites. This could confirm that the removal of rice straw extractives improved the adhesion between rice straw and the HDPE matrix. The stress was more efficiently transferred from the HDPE matrix to the rice straw. CW removed the least extractives of rice straw (Table 1) and the surface of CW-RS could retain more impurities than HW-RS and AL-RS. Therefore, the adhesion between CW-RS and the HDPE matrix was poorer than HW-RS and AL-RS, and the *E*′ of CW/HDPE was much lower than HW/HDPE and AL/HDPE. Cellulose is the major framework component of natural fibers and provides stiffness stability for the natural fibers [19,20]. Therefore, the *E*′ magnitude of both extracted and unextracted rice straw/HDPE composites also matched the order of cellulose content of both extracted and unextracted rice straws (Table 1).

The damping factor tanδ (*E*″/*E*′) amplitude among the extracted and unextracted rice straw/HDPE composites had few differences at room temperature (Figure 2). With increasing temperature, the RS/HDPE had a higher tanδ amplitude than the extracted rice straw/HDPE composites. An interfacial poorly banded composite material tended to dissipate more energy, showing a high magnitude of damping peak in comparison to the strongly bounded interface [17]. Furthermore, the particle with high stiffness restrains the segment mobility of the matrix molecules, thus resulting in a low tanδ amplitude of the composites [21,22]. Both of these principles result in a higher tanδ amplitude of the RS/HDPE at temperatures between 50 and 120 °C.

### 3.2. Water Absorption

**Figure 3:**
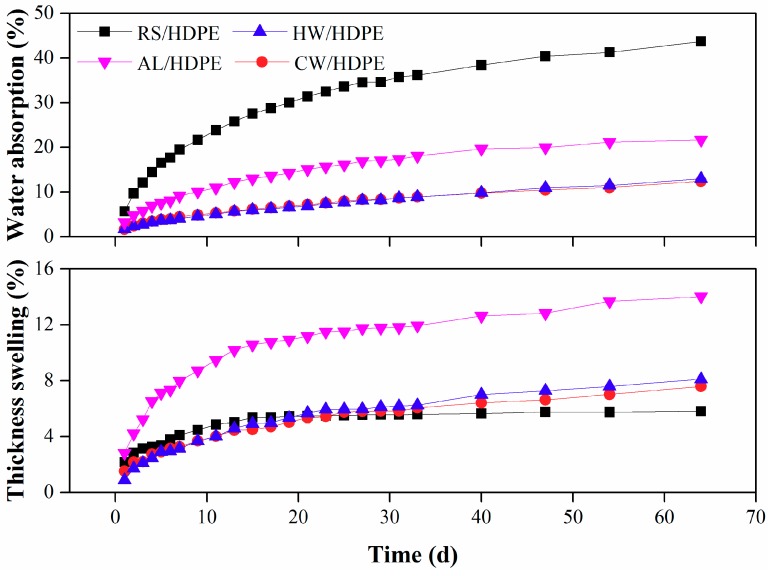
Water uptake of RS/HDPE, CW/HDPE, HW/HDPE, and AL/HDPE.

Both extracted and unextracted rice straw/HDPE composites had similar water absorption and thickness swelling tendencies, where the rates of water absorption and thickness swelling were high during the initial stage, and then gradually decreased (Figure 3). RS/HDPE absorbed more water than the other extracted rice straw/HDPE composites, but its thickness swelling was comparatively low. The water absorption of the AL/HDPE was much higher than that of CW/HDPE and HW/HDPE, and the thickness swelling of AL/HDPE was maximal. There were no evident differences between the CW/HDPE and HW/HDPE in the water absorption and thickness swelling.

The pure HDPE was naturally hydrophobic, and its water absorption was very low (<1%) [2]. The hygroscopicity of the composites was mainly due to the free hydroxyl group of rice straw. Further reasons that could lead to water absorption of the composites were the lower bonding strength between rice straw and HDPE matrix, and voids that were found in the composites (Figure 1a). No voids were found in the extracted rice straw/HDPE composites (Figure 1b–d) and the adhesion between extracted rice straw and HDPE matrix was improved. Therefore, the extracted rice straw/HDPE composites absorbed less water than RS/HDPE. Hemicelluloses and celluloses had higher capacity to absorb water [12,14], which indicated why the AL/HDPE demonstrated a more intense water absorption behavior than both the CW/HDPE and the HW/HDPE. The total contents of hemicelluloses and cellulose were very close between the CW/HDPE and the HW/HDPE (Table 1), and the water absorption behavior of the CW/HDPE and the HW/HDPE were equal.

**Figure 4:**
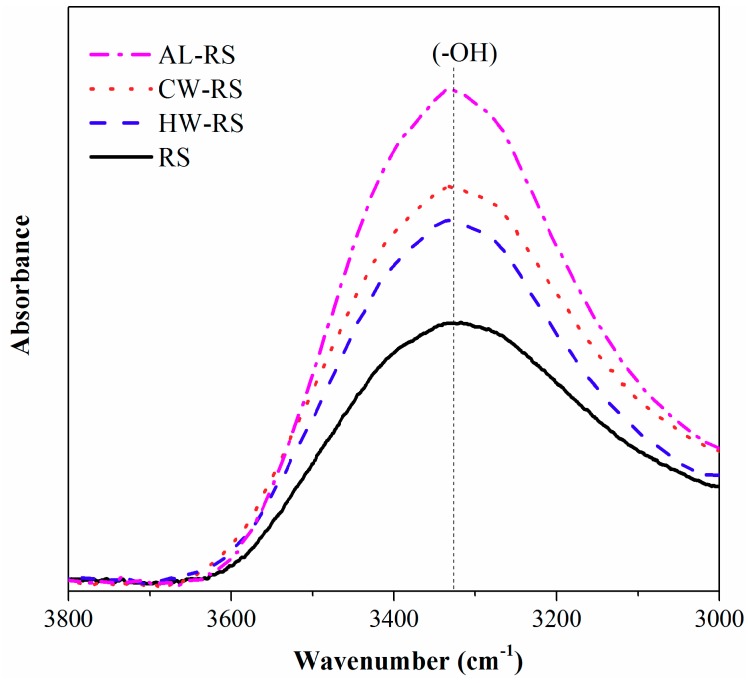
FTIR spectra of the hydroxyl groups of RS, CW-RS, HW-RS and AL-RS.

The removal of extractives of natural fiber exposed more free hydroxyl groups on the surface of the fiber (Figure 4), thus increasing the moisture absorption ability of the composites [2,23,24]. Furthermore, the content of hemicelluloses and cellulose of RS was lower than that of extracted rice straw (Table 1) and the internal voids of the RS/HDPE (Figure 1a) where water could permeate made the composite absorb the highest amount of water (a great amount of water existed in the voids) and achieved a relatively low thickness swelling. The thickness swelling of the RS/HDPE was slightly higher than the CW/HDPE and the HW/HDPE during the initial 20 days, but then decreased below that of both CW/HDPE and HW/HDPE. This phenomenon was consistent with the larger contact areas between the RS/HDPE and water than other composites, which was caused by the internal voids of the RS/HDPE. Furthermore, the thickness swelling of RS/HDPE remained stable after the water absorption of RS was close to saturation.

### 3.3. Thermal Expansion Behavior

**Figure 5:**
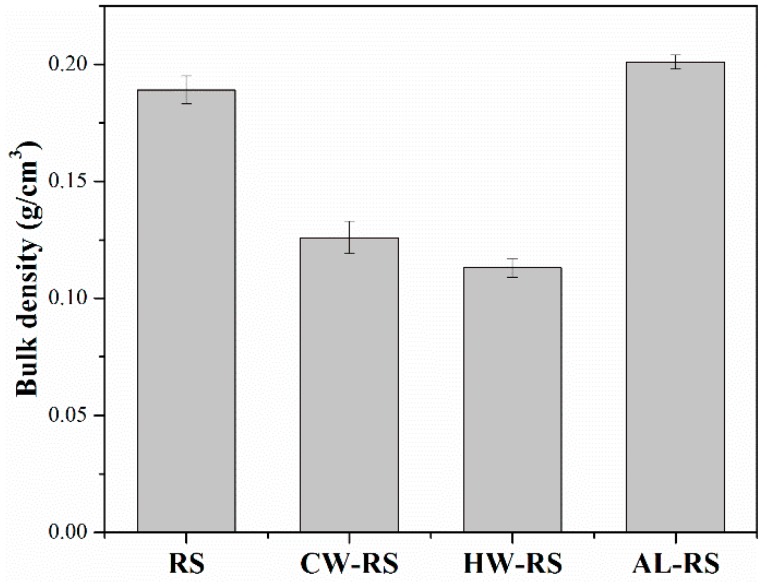
Bulk density of RS, CW-RS, HW-RS, and AL-RS.

The linear Coefficient of thermal expansion (LCTE) is a significant property for structural applications of composites; a low LCTE value is preferable for the dimensional stability of composites [25]. The LCTE of both extracted and unextracted rice straw/HDPE composites was measured in thickness during two heating stages (30 to 60 °C and −30 to 30 °C) and one cooling stage (60 to −30 °C) (Table 2). During all three stages, RS/HDPE had the lowest LCTE and AL/HDPE had the highest LCTE. The thermal expansion behavior of both CW/HDPE and HW/HDPE was very similar. The thermal expansion of the NFPCs was mainly determined by the matrix, and the natural fibers played a restrained part in NFPCs [25,26,27,28]. The inner state of the RS/HDPE was not continuous and the heat transmission of the RS/HDPE could be harder than the extracted rice straw/HDPE composites. Hence, the LCTE of the RS/HDPE was lower than that of extracted rice straw composites with a continuous inner state. The surface of the AL-RS exposed more hydroxyl groups (Figure 4) than the CW-RS and the HW-RS; therefore, the polarity of the AL-RS was stronger than that of CW-RS and HW-RS. The bulk density of AL-RS was the highest (Figure 5) in the situation of the removed extractives nearly half the weight of the rice straw (Table 1). Therefore, the agglomeration of the AL-RS that was observed could be confirmed. The agglomeration and stronger polarity made AL-RS have a less restricting effect of the non-polar HDPE than the CW-RS and the HW-RS. Thus, the LCTE of AL/HDPE exceeded those of CW/HDPE and HW/HDPE.

### 3.4. Creep and Relaxation Analysis

**Figure 6:**
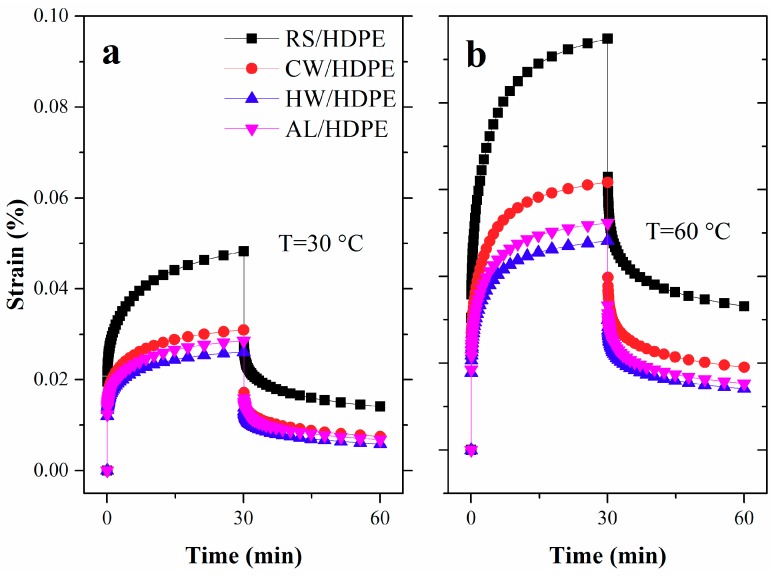
Creep strain of the RS/HDPE, CW/HDPE, HW/HDPE, and AL/HDPE during creep tests at (**a**) 30 °C and (**b**) 60 °C, respectively.

Creep is a time-dependent mechanism of material deformation and is a very important and necessary consideration for the long-term durability and reliability of material applications [29]. The creep strains of both extracted and unextracted rice straw/HDPE composites as a function of time are shown in Figure 6 at temperatures of 30 and 60 °C, respectively. At both temperatures, the RS/HDPE had the highest creep strain, followed by CW/HDEE, AL/HDPE, and HW/HDPE. The interfacial interaction between fillers and the polymer matrix played a central role in the final creep behavior of the composites [30,31]. The removal extractives of the rice straw enhanced the interfacial adhesion between the rice straw and the HDPE matrix, and significantly improved the creep properties of the rice straw/HDPE composites. The adhesion between the CW-RS and the HDPE matrix was less than that between HW-RS and AL-RS. Therefore, the creep properties of CW/HDPE were poorer than the HW/HDPE and the HW/HDPE. Moreover, the well-dispersed fillers in the matrix were beneficial for stress transfer, which resulted in reduced deformation [32]. The aggregation of AL-RS had unfavorable effects on its dispersion in the HDPE matrix, which could be the reason why the creep behavior of AL/HDPE was worse than that of HW/HDPE. The degrees of creep and residual deformation of both extracted and unextracted rice straw/HDPE composites tested at 60 °C far exceed the creep behavior of the composites at 30 °C, due to the stronger molecular motions of HDPE chain segments [28].

**Figure 7:**
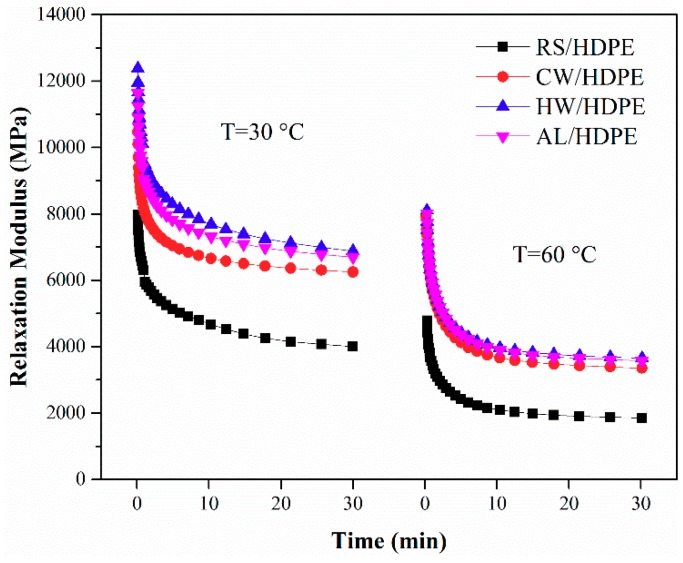
Stress relaxation of the RS/HDPE, CW/HDPE, HW/HDPE, and AL/HDPE at 30 and 60 °C, respectively.

Stress relaxation is a time-dependent material behavior that can be observed to decrease in response to the same amount of strain generated in the structure. The relaxation modulus of both extracted and unextracted rice straw/HDPE composites were measured at 30 and 60 °C for 30 min (Figure 7). The same relaxation modulus variation tendencies of extracted and unextracted rice straw/HDPE composites were observed at both temperatures. The stronger molecular motions of HDPE chain segments at higher temperature decreased the relaxation modulus of the composites. The relaxation modulus of the composites followed a decreasing order: HW/HDPE > AL/HDPE > CW/HDPE > RS/HDPE. The removal extractives of rice straw significantly improved the relaxation modulus of the resulting rice straw/HDPE composites. The difference in the relaxation modulus of both extracted and unextracted rice straw/HDPE composites could also be explained by both the interfacial interaction and filler dispersion of the composites.

## 4. Conclusions

The CW, HW and AL methods removed different amounts of extractives of rice straw. The dimensional stability (water uptake and thermal expansion), dynamic mechanical properties, creep, and stress relaxation of both extracted and unextracted rice straw/HDPE composites were investigated. The removal of extractives of rice straw decreased the water absorption of the resulting rice straw/HDPE composites; however, it increased the thickness swelling of the AL/HDPE and increased the thermal expansion of the resulting rice straw/HDPE composites. Dynamic mechanical analysis demonstrated that the stiffness of the rice straw/HDPE composites increased, and the energy dissipation of the rice straw/HDPE composites decreased via removal of rice straw extractives. The removal of rice straw extractives also significantly improved the creep and stress relaxation behaviors of rice straw/HDPE composites. Based on this study and on previous works by our groups, the HW method of extraction obtained the HW/HDPE with the best comprehensive performance.

## Figures and Tables

**Table 1:** Main chemical compositions of the unextracted and extracted rice straw.

Chemical Content	CW-RS	HW-RS	AL-RS	RS
Cellulose (%)	41.84 ± 1.34	44.53 ± 0.66	58.72 ± 0.40	31.34 ± 1.24
Hemicelluloses (%)	33.38 ± 0.31	29.63 ± 3.58	23.72 ± 1.13	26.30 ± 2.33
Lignin (%)	17.31 ± 0.84	16.46 ± 0.48	1.66 ± 0.18	14.70 ± 0.77
Ash (%)	7.47 ± 0.31	7.99 ± 0.34	5.25 ± 0.10	9.89 ± 0.10
CW extractives (%)	-	-	-	13.82 ± 0.39
HW extractives (%)	-	-	-	21.16 ± 0.59
AL extractives (%)	-	-	-	49.85 ± 0.39

**Table 2:** Linear coefficient of thermal expansion (LCTE) of the RS/HDPE, CW/HDPE, HW/HDPE, and AL/HDPE measured in the thickness direction at temperatures changing from 30 to 60 °C, 60 to −30 °C, and −30 to 30 °C, respectively.

Samples	Single-LCTE (10-6 °C-1)		
	**30 → 60**	**60 → (−30)**	**(−30) → 30**
RS/HDPE	162.7	129.3	106.4
CW/HDPE	193.8	162.8	126.7
HW/HDPE	193.4	164.0	130.3
AL/HDPE	200.3	179.0	151.2

## References

[1] Sanjay M.R., Madhu P., Jawaid M., Senthamaraikannan P., Senthil S., Pradeep S. (2018). Characterization and properties of natural fiber polymer composites: A comprehensive review. J. Clean. Prod..

[2] Sheshmani S. (2013). Effects of extractives on some properties of bagasse/high density polypropylene composite. Carbohydr. Polym..

[3] Cicala G., Tosto C., Latteri A., La Rosa A.D., Blanco L., Elsabbagh A., Russo P., Ziegmann G. (2017). Green Composites Based on Blends of Polypropylene with Liquid Wood Reinforced with Hemp Fibers: Thermomechanical Properties and the Effect of Recycling Cycles. Materials.

[4] Xie Y., Hill C.A.S., Xiao Z., Militz H., Mai C. (2010). Silane coupling agents used for natural fiber/polymer composites: A review. Compos. Part A Appl. Sci. Manuf..

[5] Celino A., Freour S., Jacquemin F., Casari P. (2013). The hygroscopic behavior of plant fibers: A review. Front. Chem..

[6] Stokke D.D., Gardner D.J. (2003). Fundamental aspects of wood as a component of thermoplastic composites. J. Vinyl Addit. Technol..

[7] Li X., Cai Z., Winandy J.E., Basta A.H. (2011). Effect of oxalic acid and steam pretreatment on the primary properties of UF-bonded rice straw particleboards. Ind. Crop Prod..

[8] Li X., Wu Y., Cai Z., Winandy J.E. (2013). Primary properties of MDF using thermomechanical pulp made from oxalic acid pretreated rice straw particles. Ind. Crop Prod..

[9] Habibi Y., El-Zawawy W.K., Ibrahim M.M., Dufresne A. (2008). Processing and characterization of reinforced polyethylene composites made with lignocellulosic fibers from Egyptian agro-industrial residues. Compos. Sci. Technol..

[10] Yao F., Wu Q., Lei Y., Xu Y. (2008). Rice straw fiber-reinforced high-density polyethylene composite: Effect of fiber type and loading. Ind. Crop Prod..

[11] El-Kassas A.M., Mourad A.H.I. (2013). Novel fibers preparation technique for manufacturing of rice straw based fiberboards and their characterization. Mater. Des..

[12] Hosseinaei O., Wang S., Enayati A.A., Rials T.G. (2012). Effects of hemicellulose extraction on properties of wood flour and wood–plastic composites. Compos. Part A-Appl. Sci. Manuf..

[13] Hosseinaei O., Wang S., Taylor A.M., Kim J.-W. (2012). Effect of hemicellulose extraction on water absorption and mold susceptibility of wood–plastic composites. Int. Biodeterior. Biodegrad..

[14] Ou R., Xie Y., Wolcott M.P., Sui S., Wang Q. (2014). Morphology, mechanical properties, and dimensional stability of wood particle/high density polyethylene composites: Effect of removal of wood cell wall composition. Mater. Des..

[15] Wise L.E., Murphy M., D’Addieco A.A. (1946). Chlorite holocellulose, its fractionation and bearing on summative wood analysis and studies on the hemicelluloses. Pap. Trade J..

[16] Lam P.S., Sokhansanj S., Shastri Y., Hansen A., Rodríguez L., Ting K. (2014). Engineering properties of biomass. Engineering and Science of Biomass Feedstock Production and Provision.

[17] Mohanty S., Verma S., Nayak S. (2006). Dynamic mechanical and thermal properties of MAPE treated jute/HDPE composites. Compos. Sci. Technol..

[18] Hristov V., Vasileva S. (2003). Dynamic Mechanical and Thermal Properties of Modified Poly(propylene) Wood Fiber Composites. Macromol. Mater. Eng..

[19] Mwaikambo L., Ansell M.P. (1999). The effect of chemical treatment on the properties of hemp, sisal, jute and kapok for composite reinforcement. Die Angew. Makromol. Chem..

[20] Kabir M.M., Wang H., Lau K.T., Cardona F. (2012). Chemical treatments on plant-based natural fibre reinforced polymer composites: An overview. Compos. Part B-Eng..

[21] López-Manchado M.A., Biagitti J., Kenny J.M. (2002). Comparative study of the effects of different fibers on the processing and properties of ternary composites based on PP-EPDM blends. Polym. Compos..

[22] Mu B., Wang H., Hao X., Wang Q. (2018). Morphology, Mechanical Properties and Dimensional Stability of Biomass Particles/High Density Polyethylene Composites: Effect of Species and Composition. Polymers.

[23] Kim J.W., Harper D.P., Taylor A.M. (2009). Effect of extractives on water sorption and durability of wood-plastic composites. Wood Fiber Sci..

[24] Sheshmani S., Ashori A., Farhani F. (2012). Effect of extractives on the performance properties of wood flour-polypropylene composites. J. Appl. Polym. Sci..

[25] Singh S., Mohanty A. (2007). Wood fiber reinforced bacterial bioplastic composites: Fabrication and performance evaluation. Compos. Sci. Technol..

[26] Nakagaito A.N., Yano H. (2008). The effect of fiber content on the mechanical and thermal expansion properties of biocomposites based on microfibrillated cellulose. Cellulose.

[27] Wu Q., Chi K., Wu Y., Lee S. (2014). Mechanical, thermal expansion, and flammability properties of co-extruded wood polymer composites with basalt fiber reinforced shells. Mater. Des..

[28] Hao X., Zhou H., Xie Y., Mu H., Wang Q. (2018). Sandwich-structured wood flour/HDPE composite panels: Reinforcement using a linear low-density polyethylene core layer. Constr. Build. Mater..

[29] Yang J., Zhang Z., Friedrich K., Schlarb A.K. (2007). Creep Resistant Polymer Nanocomposites Reinforced with Multiwalled Carbon Nanotubes. Macromol. Rapid Commun..

[30] Wang X., Gong L.-X., Tang L.-C., Peng K., Pei Y.-B., Zhao L., Wu L.-B., Jiang J.-X. (2015). Temperature dependence of creep and recovery behaviors of polymer composites filled with chemically reduced graphene oxide. Compos. Part A Appl. Sci. Manuf..

[31] Acha B.A., Reboredo M.M., Marcovich N.E. (2007). Creep and dynamic mechanical behavior of PP–jute composites: Effect of the interfacial adhesion. Compos. Part A Appl. Sci. Manuf..

[32] Bledzki A.K., Faruk O. (2004). Creep and impact properties of wood fibre–polypropylene composites: Influence of temperature and moisture content. Compos. Sci. Technol..

